# Pitting optic flow, object motion, and biological motion against each other

**DOI:** 10.1167/jov.20.8.18

**Published:** 2020-08-17

**Authors:** Krischan Koerfer, Markus Lappe

**Affiliations:** Institute for Psychology and Otto Creutzfeldt Center for Cognitive and Behavioral Neuroscience, University of Muenster, Muenster, Germany

**Keywords:** visual perception, optic flow, heading perception, object segmentation, biological motion

## Abstract

Heading estimation from optic flow is crucial for safe locomotion but becomes inaccurate if independent object motion is present. In ecological settings, such motion typically involves other animals or humans walking across the scene. An independently walking person presents a local disturbance of the flow field, which moves across the flow field as the walker traverses the scene. Is the bias in heading estimation produced by the local disturbance of the flow field or by the movement of the walker through the scene? We present a novel flow field stimulus in which the local flow disturbance and the movement of the walker can be pitted against each other. Each frame of this stimulus consists of a structureless random dot distribution. Across frames, the body shape of a walker is molded by presenting different flow field dynamics within and outside the body shape. In different experimental conditions, the flow within the body shape can be congruent with the walker's movement, incongruent with it, or congruent with the background flow. We show that heading inaccuracy results from the local flow disturbance rather than the movement through the scene. Moreover, we show that the local disturbances of the optic flow can be used to segment the walker and support biological motion perception to some degree. The dichotomous result that the walker can be segmented from the scene but that heading perception is nonetheless influenced by the flow produced by the walker confirms separate visual pathways for heading estimation, object segmentation, and biological motion perception.

## Introduction

Optic flow, the visual motion produced by a moving observer, allows estimation of one's self-motion and enables safe locomotion through the environment (Gibson, 1950; Warren, 1998; Lappe, Bremmer, & van den Berg, 1999). One mathematical description of optic flow is as a vector field providing the velocity vector at each x and y position of the retina. Given the three-dimensional (3-D) translation of the observer, the 3-D rotation of the eyes, and the distances of the objects in the scene at each retinal position, and assuming that the entire scene is rigid, the optic flow can be calculated (Longuet-Higgins & Prazdny, 1980; Heeger & Jepson, 1992). The inverse task, that is, using available optic flow information on the retina to estimate the translational direction of self-motion, is called heading estimation and is a crucial skill for navigation. Many algorithms (Longuet-Higgins & Prazdny, 1980; Heeger & Jepson, 1992; Raudies & Neumann, 2012) and neurocomputational models (Perrone, 1992; Lappe & Rauschecker, 1993; Beintema & van den Berg, 1998; Grossberg, Mingolla, & Pack, 1999; Layton, Mingolla, & Browning, 2012) have been proposed for this task. Psychophysical studies showed that humans are capable of heading estimation from optic flow within 1 to 2 degrees of error (Warren & Hannon, 1988; Warren, Morris, & Kalish, 1988; Cutting, Springer, Braren, & Johnson, 1992). This holds true even in the presence of artificial or real eye movements (Royden, Banks, & Crowell, 1992; Warren & Hannon, 1990; Royden, Crowell, & Banks, 1994; Li & Warren, 2000) and with noisy optic flow fields (Warren, Blackwell, Kurtz, Hatsopoulos, & Kalish, 1991; van den Berg, 1992).

If the scene is not rigid, for example, if an object moves independently through the environment, heading estimates become biased (Warren & Saunders, 1995; Royden & Hildreth, 1996). The bias depends on direction, speed, size, and position of the object. Surprisingly, the direction of the heading bias can, in some conditions, be in the same direction as the object motion and, in other conditions, in the opposite direction. For example, the heading bias is in the direction of object motion if the object moves purely laterally and keeps a fixed distance to the observer (Royden & Hildreth, 1996; Li, Ni, Lappe, Niehorster, & Sun, 2018). In contrast, the heading bias is often opposite to the direction of object motion if the object motion has an additional motion-in-depth component toward the observer (Warren & Saunders, 1995; Layton & Fajen, 2016a, 2016b; Li et al., 2018). The magnitude of the bias was found to depend on the visual size of the object as well as the speed ratio between the object motion and the background flow. A big fast object close to the focus of expansion results in a greater bias than a small slow object further away (Warren & Saunders, 1995; Royden & Hildreth, 1996; Layton & Fajen, 2016a, 2016b; Li et al., 2018). Biases in the direction of purely lateral object motion and against the direction of object motion for approaching objects can be reproduced in a computational model that estimates heading based on the pooled retinal motion field under the assumption of a rigid scene (Li et al., 2018). In this model, the retinal motion field areas distorted by the moving object are not treated differently from the areas of the surrounding flow and contribute equally to heading estimation. This suggests that the human heading perception system does not segment or identify the object motion but indiscriminately combines all motion vectors in the visual field for heading analysis.

In ecological conditions, independent motion in the environment occurs predominantly when other people move. The visual motion pattern experienced when one looks at a moving person has been termed biological motion and can be represented by a point-like walker whose joint positions are displayed by single points (Johansson, 1973). Each point provides information about the position of the joint at each instance in time and a motion vector similar to the optic flow. The position information supports the recognition of body posture and the perception of biological motion by analysis of posture change over time (Lange & Lappe, 2006; Theusner, de Lussanet, & Lappe, 2014). Motion vectors, in contrast, are not important for biological motion perception, as was shown in studies in which motion vectors were removed by limiting the lifetime of the points (Beintema & Lappe, 2002; Beintema, Georg, & Lappe, 2006). Two common psychophysical tasks for analyzing the perception of biological motion ask for the facing and articulation of the point-light walker (Cutting, 1981; Beintema & Lappe, 2002; Beintema et al., 2006; McKay, Simmons, McAleer, & Pollick, 2009; Reid, Brooks, Blair, & van der Zwan, 2009; Lu, 2010; Thirkettle, Scott-Samuel, & Benton, 2010; Theusner, de Lussanet, & Lappe, 2011; Masselink & Lappe, 2015). Facing refers to the orientation of the body and measures posture identification at a single point in time, while articulation refers to the complex movement of the limb segments relative to each other over time and measures the identification of posture change.

Optic flow and biological motion combine when an observer moves through a scene populated by other walkers. These walkers produce independent motion and corresponding flow disturbances. Unlike nonbiological rigid objects, the articulation pattern during walking contains information about the direction and speed of the walker's movement (Giese & Lappe, 2002; Fujimoto & Sato, 2005; Masselink & Lappe, 2015; Thurman & Lu, 2016). Yet, recent experiments suggested that this information is not used for heading detection (Riddell & Lappe, 2017, 2018; Riddell et al., 2019). A walker traversing a scene produces heading bias much like nonbiological independently moving objects (Riddell et al., 2019). When the scene consists solely of an approaching point-light walker, heading estimates are consistent with the vector sum of the self-motion of the observer and the approaching motion of the walker (Riddell & Lappe, 2017). Thus, heading estimation was based indiscriminately on the local motion vectors in the visual field without separating walker motion from self-motion. Interestingly, when the same participants were shown the same stimuli but asked to determine whether the visual motion pattern combined self-motion and biological motion, they were easily able to correctly answer the task (Riddell & Lappe, 2017). This suggests that heading perception does not take biological motion into account even though biological motion perception can discriminate biological motion from self-motion. In the present article, we aim to further clarify the relationship between the heading perception system and the biological motion perception system.

**Figure 1.**
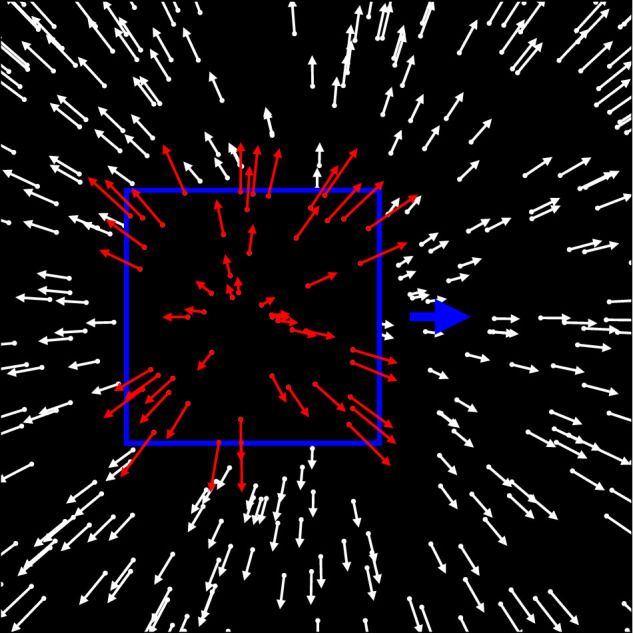
Illustration of the independent object motion stimulus as used by Warren and Saunders (1995) and Layton and Fajen (2016b). The flow field simulates heading through a cloud of dots. The blue rectangle outlines the independently moving object. The object moves to the right and toward the observer and occludes the focus of expansion of the background flow (white arrows). The local optic flow within the object (red arrows) is the resulting combination of the flow due to the observer movement and the flow due to the object motion. Thus, the distortion of the local optic flow and the motion of the object's boundary are coupled. In the original stimulus, all dots had the same color (white) and the outline of the rectangle was not shown.

**Figure 2.**
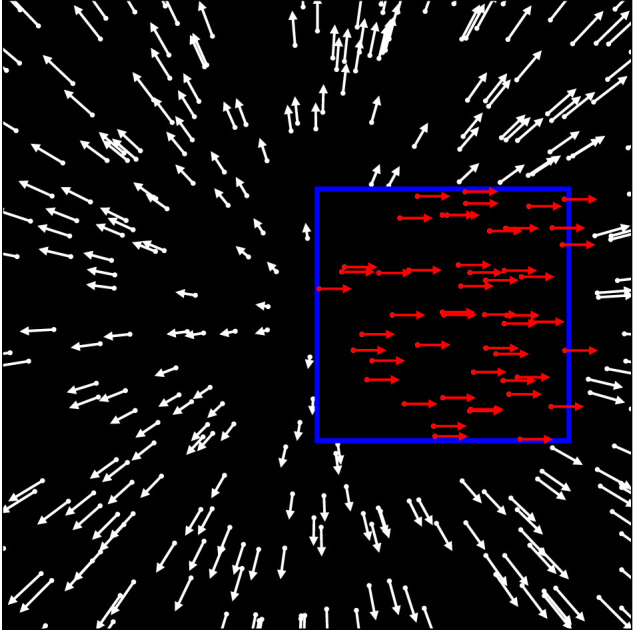
Illustration of one of the independent object motion stimulus as used by Li et al. (2018). The flow field simulates heading through a cloud of dots. The blue rectangle outlines the area in which the flow of the independently moving object is shown. The flow in this area is uniformly to the right. The area itself does not move and stays at its position, that is, forming a static object boundary. Thus, the local optic flow is decoupled from the impact of the motion of the object.

When a walker or an object moves through a scene, it creates two cues to their scene-relative motion. The first is the motion of the boundary of the object that covers and uncovers background scene elements. We will refer to this as the object's motion. The second is the flow within the boundaries of the object, which, when embedded into a self-motion flow field, presents a local distortion of the flow field's structure. With the exception of Li et al. (2018), previous studies that investigated heading perception in the presence of a moving object used objects defined by a pattern of dots that collectively moved across the screen (Figure 1) (Warren & Saunders, 1995; Royden & Hildreth, 1996; Layton & Fajen, 2016a, 2016b; Li et al., 2018). All dots moved according to the optic flow caused by the self-motion of the observer. Direction and speed of the motion of each dot are indicated by the arrows in the figure. However, the dots that made up the object, the ones that are within the blue rectangle in the figure, had an additional motion component due to the motion of the object. In this case, object motion and local optic flow distortion are inherently coupled. The same is true for the studies by Riddell et al. (2017, 2018, 2019), which presented biological motion walkers for which the motion of the point lights was fully coupled to the motion of the walker. Li et al. (2018), on the other hand, presented a similar stimulus, but with a key difference (Figure 2). The dots of a random dot pattern moved according to the self-motion of the observer, but dots within a small area of the visual field moved differently in order to display the distorted local optic flow that a moving object would introduce (red arrows within the blue rectangle in the figure). However, the area itself was static and did not move at all. In this case, the local optic flow is decoupled from the object motion, and the impact of the distortion of the local optic flow on heading estimation can be analyzed in isolation. However, since the object borders were stationary in this stimulus, any impact of the object's motion on heading estimation could not be analyzed independently.

**Figure 3.**
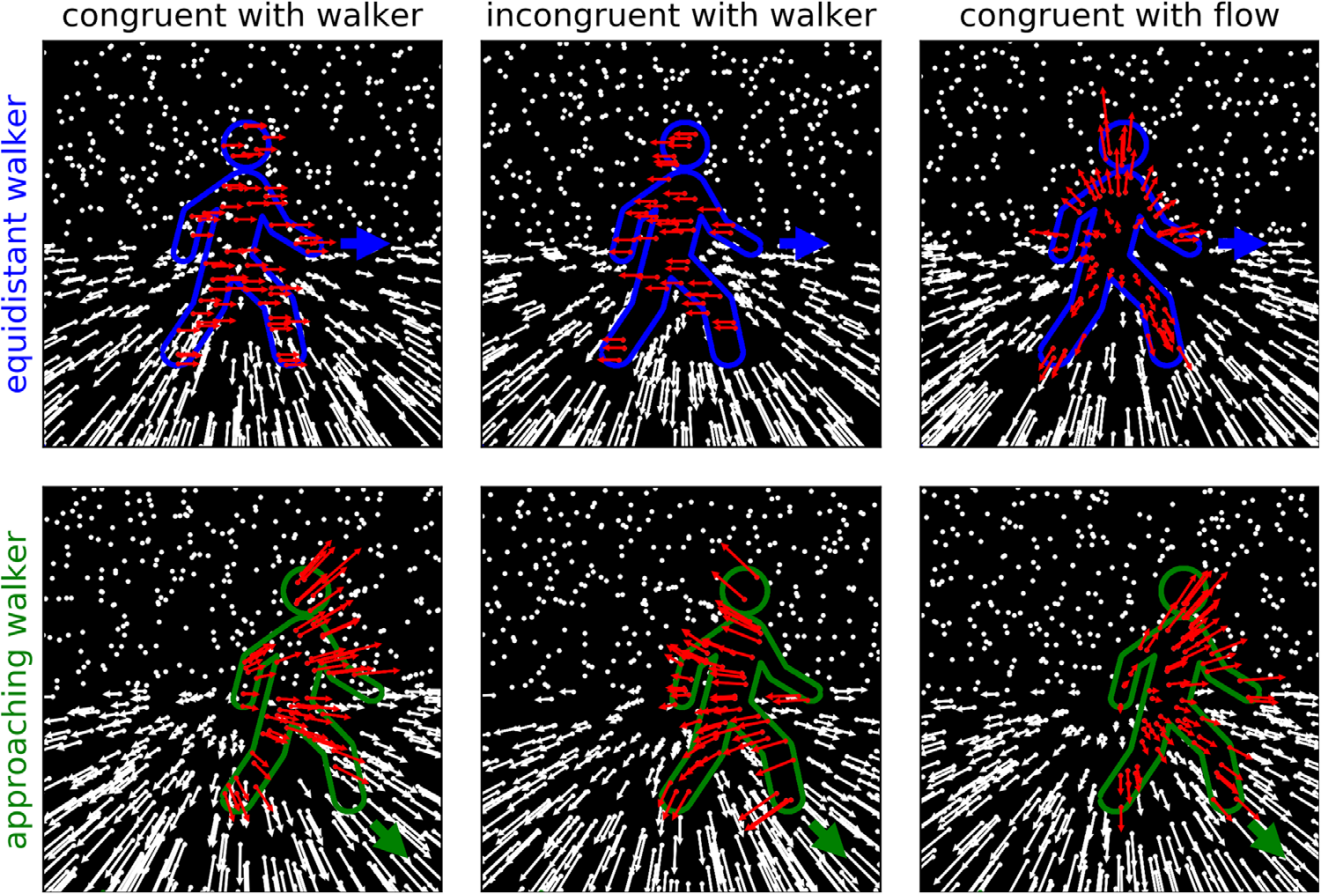
Scheme of the three equidistant and three approaching walker stimuli with different local optic flow. The background scene consists of a ground plane covered with random dots and a sky above, also covered with random dots. White arrows illustrate the optic flow due to observer motion. Dots in the sky are presumed infinitely far away and produce no flow. The blue/green outline (not visible in the actual stimulus) indicates the body of the walker. As the walker moves in the direction of the blue/green arrow, its body is reshaped and translated, simulating limb articulation and forward movement. The panels on the left depict local flow congruent with the walker's motion. Dots within the walker's body move along with the walker's motion. The center panels depict local flow incongruent with the motion of the walker. For the equidistant walker, all dots within the walker area move uniformly to the left with the same speed as the walker moves to the right. For the approaching walker, the dots move as if the lateral component of the walker motion was not to the right but instead to the left. The right panels show a condition in which the local flow is congruent with the global optic flow. The dots inside the walker move as if they were static parts of the scene located at the height above ground of the respective body part of the walker. Since the walker is closer to the observer than the parts of the ground plane and sky that its body covers, the direction of motion of the dots within the walker is fully linked to the observer's heading but their speed makes the walker stand out from the background.

In the present study, we present combinations of optic flow and independent biological motion in which the object motion and the flow distortion are fully decoupled and in which the flow distortion can be congruent or incongruent with either self-motion or object motion. To this end, we developed a novel stimulus in which a walker shape is molded solely by differences in the optic flow field. The stimuli are depicted in Figure 3. Every single frame of the stimulus consists of a random dot distribution, presenting no structural information. The motion of the dots and thus the optic flow are consistent with an observer walking across a ground plane (white arrows in the figure). However, within the body of a walker, the optic flow vectors are altered from those of the background scene (red arrows in the figure), thus allowing the presentation of an object shape. By moving and reshaping this area from frame to frame, biological motion can be presented independently from the local optic flow field within the shape.

We conducted three experiments with these stimuli: a heading experiment as discussed above to investigate the heading bias and a facing and an articulation experiment. The latter two experiments use typical tasks of biological motion perception. They test whether the human shape and motion can be perceived from these novel biological motion stimuli. This is important to qualify any specific findings of the heading experiment. In order to investigate whether the heading bias stems from optic flow or from the walker moving through the scene, it is critical to show that participants perceive the walker. Moreover, these experiments aimed to see whether the walker's shape could be segmented based on optic flow field cues. The stimuli simulate observer motion over a ground plane with a sky of random dots infinitely far away and an additional walker that could traverse the scene with a constant distance from the observer or approach the observer at an angle. The dots within the walker shape could move congruent with the walker motion, incongruent with the walker motion, or congruent with the global flow.

## Method

### Participants

The sample consisted of 12 participants (5 female and 7 male) with age ranging from 23 to 35 naive to the purpose of this study. All had normal or corrected-to-normal vision. The number of participants was chosen to allow a counter balance order of the three experiments. The study was approved by the Ethics Committee of the Department of Psychology and Sport Science of the Westfälische Wilhelms Universität, Münster, Germany. Participants gave written consent to participate in the study.

### Setup

The stimuli were created with Python 3.5 and SciPy 1.1.0 on an Apple MacBook Pro 2017 equipped with a Radeon Pro 560 graphic card with 4096 MB memory. The frames of the stimuli were precomputed and saved as PNG files because online rendering was not fast enough. The files were projected with the same MacBook, Python 3.5, Psychopy 3.0, and a VDC Display Systems Marquee 8500 projector onto a 224×185-cm backlit screen with 800×600 pixels and 60-Hz refresh rate.

Participants sat in a chair central in front of the screen at a 1-m distance. They used the left/right and up/down keys of a keyboard for the facing and articulation task, respectively, and a mouse pointer and left click of a two-button laser mouse on a wooden plate for the heading task.

### Stimuli

The stimulus simulated walking over a ground plane with a static sky above. The stimulus showed the central view from an artificial camera 1.8m above the plane. Sky and plane were represented by white pixel-sized dots on a black background. Those dots were placed randomly with a density of $1.4694\;\text{dots/deg}{}^{2}$, so that any single frame showed no spatial feature of the plane or sky and appeared as white noise. Motion of the observer was simulated by updating the position of the dots on the plane according to the optic flow calculated for that observer motion. Dots in the sky remained static. Dots had a limited lifetime such that every dot had a probability of 0.15 per frame to be replaced with a dot at a random position. All dots that left the screen during simulated observer movement were spawned back at a random position. This ensured a steady dot density over time and a white noise distribution in every single frame. The observer traveled with 1.5m/s on a straight line over the plane, either toward the center of the screen or 5° or 10° to the left or right, resulting in five possible heading directions. The “no-walker” condition simply presented these stimuli. The stimulus duration was 1.7s.

In the other conditions, a walker was placed on the ground plane. The walker was constructed from a motion-capture recording of a walking human actor (de Lussanet et al., 2008). The recording consisted of the motion of 18 points representing the head, shoulders, elbows, wrists, hands, hips, knees, ankles, and tips of the feet. To create the body shape of the walker, each of those points was assigned a circular area of radius $\sqrt{0.02}\;\text{m}$ in which dots could be presented. Given the dot density of $1.4694\;\text{dots/deg}{}^{2}$ and a walker distance of 1.5m, 135 dots were inside each of these circular areas on average. The collective of these areas, which changed in every frame due to the articulation of the limbs, formed the body of the walker. The walker walked with a speed of 1.2m/s and took 1.1333s or 68 frames to complete one walking cycle. The stimulus duration was 1.7s, resulting in 1.5 walking cycles.

We tested two different walker paths, following earlier studies on independent object motion (Warren & Saunders, 1995; Royden & Hildreth, 1996; Li et al., 2018). In the “equidistant” condition, the walker started 1.02m left or right from the center of the display, then crossed the observer's path, and finished 1.02m on the other side of the center of the display. The walker's facing was $\pm 90^{\circ }$ and the articulation matched the lateral translation. During this movement, the walker was shifted in depth with the observer movement such that it kept a fixed distance of 1.5m from the observer while walking. This is similar to the paradigm of Royden and Hildreth (1996) and to the lateral motion condition of Li et al. (2018). Based on the results of those studies, we expect a bias in the direction of the local optic flow within the equidistant walker shape. In the “approaching” condition, the walker started in the center of the display, 5m in front of the observer, and walked along a $45^{\circ }$ tilted path to the left or right, facing in walking direction and articulating according to the translation. In this condition, the walker moved in depth toward the observer, similar to the paradigm of Warren and Saunders (1995) and the motion-in-depth condition of Li et al. (2018). Based on the results of those studies, we expect a bias against the direction of the local optic flow within the approaching walker shape.

We tested three different motion patterns of the dots within the walker shape. For the “congruent-with-walker” condition, the circular areas that formed the body of the walker were filled with white pixel-sized dots with the same density as the background flow. These dots were locked in place relative to the center of each respective circular area and thus moved with the walker. For the “incongruent-with-walker” condition, the body of the walker was superimposed on the scene and any dot of the plane or the sky that fell within the body area was moved with a local motion vector inverse to the lateral motion of the walker (i.e., same speed but opposite lateral component). Thus, in the equidistant condition in which the walker moved rightward but kept a fixed distance from the observer, the dots simply moved leftward. In the condition in which the walker approached the observer while walking from the center to the right, the dots moved as if the walker was still approaching but moving from the right to the center. For the “congruent-with-flow” condition, any dot of the plane or the sky that fell within the body area was treated as if it corresponded to a static object at the depth position of the walker. Thus, those dots moved as if they belonged to the background flow but with increased speed because the walker was closer to the observer than the background.

For the incongruent-with-walker and congruent-with-flow conditions, dots often crossed the walker outline as the dot motion and the walker shape motion were not aligned. When dots crossed the outline, they were captured such that the speed and direction of their motion matched the intended optic flow at their current position.

In the facing and articulation tasks of Experiments 2 and 3, observer motion was straight ahead, walkers faced left or right, and walker motion presented four different cases. Articulation was either forward or backward, the latter generated by inverting the order of the frames in the walking cycle. Moreover, translation of the walker could either match the articulation or be in opposite direction, resulting in either normal or “moonwalking” stimuli. The presentation duration of each stimulus was 1.7s, corresponding to 1.5 walking cycles and ensuring that the walker's posture in the final frame was the same in all conditions.

### Tasks

In the heading task (Experiment 1), participants were asked to report their perceived direction of heading. During stimulus presentation, they had to fixate a central white fixation cross (10 by 10 pixels). After the stimulus presentation, the last frame remained visible and a red vertical line (25 pixels tall and 5 pixels wide) appeared. The participant moved this line along the horizontal axis with the mouse to the location of the perceived heading. Left mouse click saved the current position as heading response and started the next trial.

In the facing task (Experiment 2), participants were asked to report the facing direction (left or right) of the walker. Responses were registered by button presses. No fixation cross was present, and there was no visual feedback to the responses. Observer motion was always straight ahead and walker motion presented four different cases. Articulation was either forward or backward, the latter generated by inverting the order of the frames in the walking cycle. Moreover, translation of the walker could either match the articulation or be in opposite direction, resulting in either normal or “moonwalking” stimuli.

In the articulation task (Experiment 3), stimuli were the same as in the facing task, but participants were asked to report whether the walker's limb motion showed forward or backward walking, independent of the translation of the walker. The presentation duration of each stimulus was 1.7s, corresponding to 1.5 walking cycles and ensuring that the walker's posture in the final frame was the same in all conditions.

### Procedure

The participants were randomly assigned to one of six groups in order to counterbalance the order of the three experiments with heading task, facing task, and articulation task, respectively. Participants could choose to do all experiments in succession or to split them to different days.

Before the experiment, participants received instructions and familiarized themselves with stimuli and tasks in a few practice trials. For the heading task, a scene without walkers was shown twice for each of the five possible heading directions in random order. Participants did not receive any feedback about their performance. For the heading and articulation tasks, practice stimuli consisted of a walker in front of a black background. Each case of forward walking, backward walking, forward moonwalking, and backward moonwalking was shown twice in random order. Participants did not receive any feedback on their performance.

The heading experiment consisted of 5*7*2*4=280 trials. There were five heading directions and seven walker conditions: no walker, equidistant congruent-with-walker, equidistant incongruent-with-walker, equidistant congruent-with-flow, approaching congruent-with-walker, approaching incongruent-with-walker, and approaching congruent-with-flow. Walkers could walk to the right or the left (for the no-walker condition, the same stimulus was presented in either case). There were four repetition blocks. Within one block, the walker conditions were ordered randomly and all the heading direction conditions and left/right conditions were shown in random order while keeping the walker condition fixed for these 10 trials. Between blocks, participants could shortly rest or continue at own will.

The facing and articulation experiments used the same stimuli and had 4*6*2*4=192 trials. There were four articulation conditions (normal forward/backward and moonwalking forward/backward). The six walker conditions were equidistant congruent-with-walker, equidistant incongruent-with-walker, equidistant congruent-with-flow, approaching congruent-with-walker, approaching incongruent-with-walker, and approaching congruent-with-flow. The walker could again move to the right or left. There were four repetition blocks. Within one block, the walker conditions were ordered randomly and the articulation conditions and the left/right conditions were shown in random order while keeping the walker condition fixed for eight trials. Between blocks, participants could shortly rest or continue at own will.

## Results

### Experiment 1: Heading bias

Experiment 1 measured heading bias in the different walker conditions. We first analyzed the “no-walker” condition in order to verify that heading estimation can be performed on the newly developed stimulus. Since the stimulus presents a high density of dots with short lifetime, it might happen that a vanishing dot and an appearing dot in close proximity produce spurious motion signals and add considerable noise to the optical flow. The “no-walker” condition provides a baseline of heading estimation performance under these conditions.

A linear regression on the accumulated heading estimation data of the 12 participants for the five simulated heading directions yielded a regression coefficient between simulated and perceived heading of 0.548 with a 95% confidence interval of [0.494,0.602]. The intercept was $-0.111^{\circ }$ with a 95% confidence interval of $\left[-0.495^{\circ },\;0.274^{\circ }\right]$. The regression coefficient of 0.548 indicates a heading bias toward the center. Such a center bias has often been reported in earlier studies (Warren et al., 1988; Warren & Saunders, 1995; Royden, 1996) and might have been amplified in our data by missing heading cues in the sky and the high noise in the optic flow. Nonetheless, with a regression coefficient of 0.548[0.494,0.602] of simulated and perceived heading, the heading estimation was sufficiently accurate to justify further investigation of the other conditions. Importantly, the intercept was not significantly different from $0^{\circ }$ and, thus, no left or right bias occurred. Therefore, in further analysis, we collapsed equivalent conditions with mirror symmetry and report biases relative to the direction of the walker motion. For example, the stimulus presenting an artificial heading of $-10^{\circ }$ and a walker going to the left was treated as equal to the stimulus presenting an artificial heading of $10^{\circ }$ and the same walker going to the right. A positive heading bias denotes a bias in walker motion direction and a negative bias in opposite direction.

**Figure 4.**
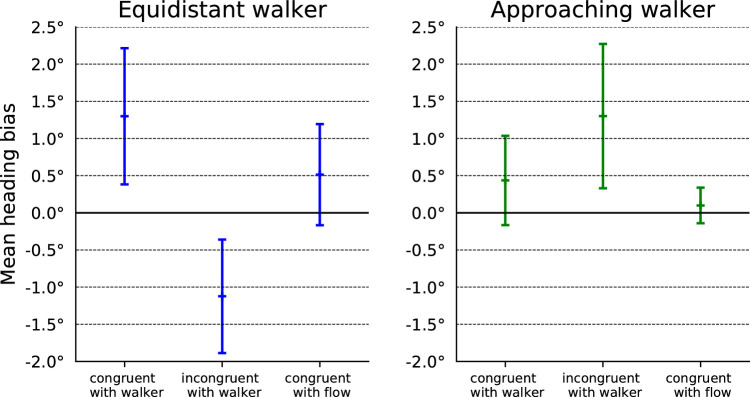
Results of Experiment 1: heading bias in walker motion direction for the equidistant walker on the left and for the approaching walker on the right. The graphs show the mean over all participants and the 95% confidence interval based on the local optic flow of the walker being congruent with walker motion, incongruent with walker motion or congruent with the optic background flow due to the heading.

To analyze the influence of the walkers and their different local optical flow distortions, the heading estimations of all heading directions were averaged for every participant and walker condition. These data were then further averaged over all participants to yield a mean heading bias and a 95% confidence interval for each condition. The results are shown in Figure 4. A one-way repeated-measures analysis of variance with Greenhouse-Geisser correction showed a significant influence of the local optic flow condition (p=0.005). Planned comparison *t* tests were conducted to analyze the conditions relevant to our hypothesis as described below.

In the equidistant condition, previous research had shown that the bias is directed in the direction of the independent object motion (Royden & Hildreth, 1996; Li et al., 2018). If this bias is driven by the local optic flow within the object shape, we expect it to be directed against walker motion in the incongruent-with-walker condition. Second, we expect the bias to vanish in the congruent-with-flow condition. In contrast, if the bias were driven by the object motion (i.e., the motion of the shape of the walker), we expect the same bias in the direction of walker motion in all three conditions.

The congruent-with-walker condition yielded a significant (one-sample *t* test, p=0.016) bias in walker direction of $1.299^{\circ }$ with a 95% confidence interval of $\left[0.383^{\circ },\;2.215^{\circ }\right]$, consistent with earlier results (Royden & Hildreth, 1996; Li et al., 2018). The incongruent-with-walker condition yielded a mean bias of $-1.123^{\circ }$ with a 95% confidence interval of $\left[-1.886^{\circ },\;-0.3596^{\circ }\right]$, that is, a bias against motion direction (one-sample *t* test, p=0.013). This bias had the same magnitude but opposite direction as the bias of the congruent-with-walker condition. This is strong evidence, that the heading bias depends on the local optic flow, which had indeed the same strength but opposite sign in the two conditions. The congruent-with-flow condition showed no significant bias (one-sample *t* test, p=0.160), also consistent with the predictions of the dependency on local optic flow.

In the approaching condition, the bias in previous studies was usually directed against the direction of independent object motion (Warren & Saunders, 1995; Li et al., 2018) but varied with object distance, size, angle of approach, and speed (Warren & Saunders, 1995; Layton & Fajen, 2016a, 2016b; Li et al., 2018). If the bias is driven by local optic flow within the object shape, we expect it to be directed in walker motion in the incongruent-with-walker condition. Moreover, we expect no bias in the congruent-with-flow condition. If the bias were driven by the object motion (i.e., the motion of the shape of the walker), we instead expect a bias against the direction of walker motion in all three conditions.

In the congruent-with-walker condition, our data did not show any significant bias (one-sample *t* test, p=0.175). This is different from earlier studies (Warren & Saunders, 1995; Layton & Fajen, 2016a, 2016b; Li et al., 2018) but might be explained by differences in object distance, size, angle of approach, or speed between or study and those previous studies. The incongruent-with-walker condition, however, yielded the predicted bias in walker direction of $1.301^{\circ }$ with a 95% confidence interval of $\left[0.331^{\circ },\;2.271^{\circ }\right]$, which was both different from zero (one-sample *t* test, p=0.021) and different from the value obtained in the congruent-with-walker condition (paired *t* test, p=0.019). Also as predicted, the congruent-with-flow condition produced no significant bias (one-sample *t* test, p=0.425).

In summary, the results of the first experiment support the role of local optic flow within the object shape.

### Experiment 2: Shape segmentation and facing discrimination of biological motion

The second experiment tested whether the walker's shape could be segmented based on optic flow field cues and whether the segmented shape could be identified as a walker. The stimuli were identical to those of Experiment 1, with the exception that the walker in half of the trials walked backward instead of forward. In combination with random leftward or rightward facing, this led to half of the trials showing normal forward or backward walking, respectively, and the other half showing their “moonwalking” counterparts. This arrangement therefore decouples facing direction, articulation, and translation and ensures that facing could be determined only if the shape of the walker could be segmented and its posture could be determined. Participants were asked to report whether the stimulus included a walker facing to the left or a walker facing to the right. Considering the small differences in the postures between walkers facing in opposite direction, this was a challenging task that required a high level of shape detail to be perceived.

**Figure 5.**
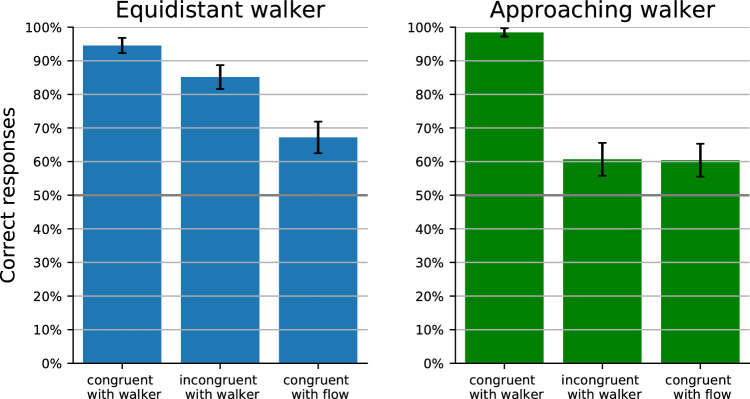
Results of Experiment 2: percentage of correct discrimination of facing direction (left vs right) for the equidistant and approaching walker in the different local optic flow conditions. Error bars show 95% confidence intervals.

The results are presented in Figure 5. The data were collapsed across all participants and all facing and articulation conditions. The 95% confidence intervals were approximated with a normal distribution. Figure 5 shows that performance was above chance level in all conditions but different in different conditions.

In the congruent-with-walker condition, participants could identify the correct facing of the equidistant walker with 94.5%±2.3% correct and of the approaching walker with 98.4%±1.2% correct. These high accuracies show that the size of the walker, the stimulus duration, and the detail of the walker shape were in general sufficient for reliable facing detection for both walker types.

In the incongruent-with-walker condition, facing perception was 85.2%±3.6% correct for the equidistant walker and 60.7%±4.9% for the approaching walker. Hence, this condition appeared more challenging but still possible. The lower accuracy might be due to difficulties in pursuing the shape as eye motion would have to be against optic flow direction. The low performance for the approaching walker might be further influenced by the greater distance of the walker at the beginning of the stimulus or the smaller difference in orientation for the two different facings.

In the congruent-with-flow condition, facing perception was 67.2%±4.7% correct for the equidistant walker and 60.4%±4.9% for the approaching walker. The equidistant is particularly challenging since the optic flow direction within the walker shape reverses direction once the walker passes the center of the screen, which might disrupt the perception of the walker.

In summary, the observation that facing discrimination performance was above chance in all conditions shows that shape segmentation based on flow field cues is possible and sufficient for facing perception of biological motion. Comparisons between conditions suggest that segmentation is easiest when the local flow within the walker shape is congruent with the walker motion, less easy when it is incongruent with the walker motion, and most difficult when it is congruent with the background flow.

### Experiment 3: Articulation discrimination of biological motion

Experiment 3 tested the perception of biological motion with an articulation task. The stimuli were the same as in Experiment 2, but participants were now asked to determine whether the walker's articulation was forward or backward. This is a harder task than facing discrimination because the articulation of a walker cannot be determined by a single posture but requires a sequence of different postures (Beintema et al., 2006; Lange & Lappe, 2006; Theusner et al., 2014). In the stimuli presented in this study, this sequence had to be derived from the evolution of the optic flow field over time.

**Figure 6.**
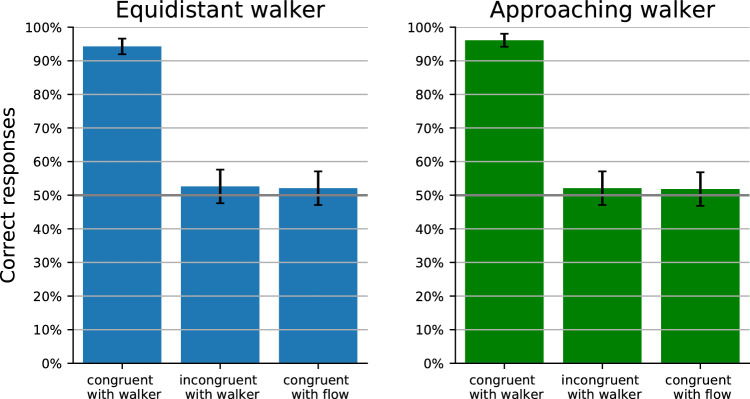
Results of Experiment 3: percentage of correct recognition of articulation direction (forward vs. backward) for the equidistant and approaching walker in the different local optic flow conditions. Error bars show 95% confidence intervals.

Results are presented in Figure 6. The data were collapsed for all participants and all facing and articulation conditions. The 95% confidence intervals were approximated with a normal distribution. In the congruent-with-walker condition, participants could easily do the task for either the equidistant walker (percent correct: 94.3%±2.3%) or the approaching walker (percent correct: 96.1%±1.9%). For the incongruent-with-walker condition and the congruent-with-flow condition, however, performance was at chance level for both walkers (equidistant incongruent-with-walker: 53.0%±5.0%; equidistant congruent-with-flow: 52.1%±5.0%; approaching incongruent-with-walker: 52.1%±5.0%; approaching congruent-with-flow: 51.8%±5.0%). In these conditions, participants appeared unable to extract sufficient information from the evolution of the optic flow field to segment different but similar postures after one another and to estimate posture change for a coherent walking cycle percept. The weak performance in these conditions is consistent with the results from Experiment 2, which had already shown that the incongruent-with-walker and the congruent-with-flow conditions supported only weaker segmentation and facing discrimination than the congruent-with-walker condition.

## Discussion

We studied optic flow and biological motion perception using novel stimuli in which the local motion of the two is fully decoupled. In these stimuli, the local flow within the body shape of the walker could be congruent with the walker's motion, incongruent with the walker's motion, or congruent with the surrounding flow field.

We found that heading estimation was simply based on the local flow within the walker shape, together with the surrounding flow, and not on the walker's motion, that is, the motion of the walker's shape boundary or its walking. This conclusion is drawn from the heading estimation bias in the equidistant conditions, which was opposite the direction of the local motion in the congruent-with-walker and the incongruent-with-walker conditions and absent in the congruent-with-flow condition, as predicted from earlier studies of heading bias (Royden & Hildreth, 1996; Li et al., 2018). Similarly, in the approaching incongruent-with-walker condition, bias was against the direction of local motion, as predicted from earlier studies (Warren & Saunders, 1995; Layton & Fajen, 2016a, 2016b; Li et al., 2018), and absent, as expected, in the congruent-with-flow condition. The approaching congruent-with-walker condition was not show the expected bias against the direction of local flow. The heading bias produced by independent object motion is known to be sensitive to the precise relation between the flow produced by the moving object and the background flow. The failure to replicate the bias against the direction of local flow might be due to the complexity of the biological motion stimulus in the present study. We are confident, however, that the lack of bias is not related to an influence of the walker's motion because the local flow within the walker shape and the walker's motion was congruent in this condition and should have added up if the motion of the walker shape also produced a bias.

Overall, the heading results corroborate earlier findings that heading is based on the pooled optic flow without segmentation of independently moving objects (Li et al., 2018) or biological motion (Riddell & Lappe, 2017; Riddell et al., 2019). This supports, on the one hand, different pathways for object segmentation and heading perception in the human brain (Rushton, Niehorster, Warren, & Li, 2018; Warren, Rushton, & Foulkes, 2012; Yu, Hou, Spillmann, & Gu, 2017; Li et al., 2018) and, on the other hand, different pathways for biological motion and heading perception (Beintema & Lappe, 2002; Lange & Lappe, 2006; Theusner et al., 2014; Mayer, Riddell, & Lappe, 2019). Different neural pathways are further supported by physiological findings. The superior temporal sulcus has been associated with biological motion perception (Beauchamp, Lee, Haxby, & Martin, 2002; Grossman et al., 2000; Grossman & Blake, 1999; Grossman, Battelli, & Pascual-Leone, 2005; Hirai, Fukushima, & Hiraki, 2003; Krakowski et al., 2011; Michels, Kleiser, de Lussanet, Seitz, & Lappe, 2009) and there is no evidence for a link with the areas specialized in optic flow processing (Morrone et al., 2000; Grossman et al., 2005; Smith, Wall, Williams, & Singh, 2006; Pitzalis et al., 2010). It has been argued that, since the heading bias produced by independent object motion is rather small, heading estimation from the pooled flow may be sufficient for safe locomotion through the environment while avoiding complex and time-consuming segmentation (Li et al., 2018).

Experiments 2 and 3 were concerned with shape segmentation and biological motion perception. Experiment 2 showed that shape segmentation was possible and can be based solely on cues in the optic flow field. Since the stimuli presented altered local flow within the walker shape while keeping all other visual properties constant, shape segmentation could not use any difference in luminance, color, density, flicker, or occlusion. Instead, the walker shape could only be segmented based on the difference in optic flow field dynamics within and outside the shape. Note that the segmentation is not a simple pop-out from an otherwise homogeneous global optic flow as the background optic flow already consists of a complex radial structure with areas of different optic flow magnitudes and directions. Neither is it a segmentation of areas inconsistent with heading as the walker segmentation was still possible in the congruent-with-flow condition, in which heading of the background flow and heading of the flow within the walker shape were identical. The segmentation might be derived from the boundaries between different optic flow dynamics, as these were always present in this study, but it is also possible that the visual system is capable of segmenting different optic flow field dynamics with a smooth transition between them. The understanding of the extent and underlying mechanism of this segmentation skill is still limited and warrants further research.

The finding that shape segmentation was possible in Experiment 2 adds further relevance to the finding of Experiment 1 that shape segmentation is not used in heading estimation and, hence, to the different pathways for heading estimation and object motion perception.

Experiment 2 furthermore showed that the segmented shape supports the recognition of a human walker and the discrimination of its facing direction. Facing discrimination was possible for all local motion conditions, confirming that it is based on motion segmentation cues in the stimulus and not on the local flow. Although facing discrimination performance was best when local flow within the body shape was congruent with the motion of the walker, the local flow direction was useless for facing discrimination because the walker could walk forward or backward, thus decoupling local flow from facing. The superior performance for the congruent-with-walker condition, therefore, suggests a superior segmentation ability in this condition.

Experiment 3 investigated whether the shape segmentation could support more complex biological motion perception, namely, the discrimination between forward and backward walking. This discrimination is more difficult because it needs information about the posture change between two successive postures and has often been used to investigate true body motion perception (Beintema et al., 2006; Lange & Lappe, 2006; Theusner et al., 2014). The results showed that discrimination between forward and backward walking was possible but only in the congruent-with-walker condition. For the other two conditions, performance was at chance, presumably because segmentation performance (as measured in Experiment 2) was not sufficient to support the analysis of posture changes in subsequent frames.

In summary, the present study shows the divisions between different processes of ecological motion perception. Differences between the local flow within a moving object and the background optic flow can be used to segment independently moving objects, even if they transform their shape over time, as walkers do. Nevertheless, heading estimation does not use the segmentation information but combines all flow, the local flow within the object shape, and the background flow, indiscriminately. Biological motion perception, on the other hand, is possible — if difficult — from the segmented nonrigid shape.

## References

[bib1] Beauchamp M. S., Lee K. E., Haxby J. V., & Martin A. (2002). Parallel visual motion processing streams for manipulable objects and human movements. *Neuron,* 34, 149–159. 11931749 10.1016/s0896-6273(02)00642-6

[bib2] Beintema J. A., Georg K., & Lappe M. (2006). Perception of biological motion from limited lifetime stimuli. *Perception & Psychophysics,* 68, 613–624. 16933426 10.3758/bf03208763

[bib3] Beintema J. A., & Lappe M. (2002). Perception of biological motion without local image motion. *Proceedings of the National Academy of Sciences, USA,* 99, 5661–5663. 10.1073/pnas.082483699 PMC122827 11960019

[bib4] Beintema J. A., & van den Berg A. V. (1998). Heading detection using motion templates and eye velocity gain fields. *Vision Research,* 38, 2155–2179. 9797976 10.1016/s0042-6989(97)00428-8

[bib5] Cutting J. E. (1981). Coding theory adapted to gait perception. *Journal of Experimental Psychology: Human Perception and Performance,* 7, 71–87.

[bib6] Cutting J. E., Springer K., Braren P. A., & Johnson S. H. (1992). Wayfinding on foot from information in retinal, not optical, flow. *Journal of Experimental Psychology: General,* 121, 41–72. 1534347 10.1037//0096-3445.121.1.41

[bib7] de Lussanet M. H. E., Fadiga L., Michels L., Seitz R. J., Kleiser R., & Lappe M. (2008). Interaction of visual hemifield and body view in biological motion perception. *European Journal of Neuroscience,* 27, 514–522. 18215244 10.1111/j.1460-9568.2007.06009.x

[bib8] Fujimoto K., & Sato T. (2005). Backscroll illusion: Apparent motion in the background of locomotive objects. *Vision Research,* 46, 14–25. 16289275 10.1016/j.visres.2005.09.027

[bib9] Gibson J. J. (1950). *The perception of the visual world*. Houghton Mifflin, Boston. Boston, MA: Houghton Mifflin.

[bib10] Giese M. A., & Lappe M. (2002). Measurement of generalization fields for the recognition of biological motion. *Vision Research,* 42, 1847–1858. 12128015 10.1016/s0042-6989(02)00093-7

[bib11] Grossberg S., Mingolla E., & Pack C. (1999). A neural model of motion processing and visual navigation by cortical area MST. *Cerebral Cortex,* 9, 878–895. 10601006 10.1093/cercor/9.8.878

[bib12] Grossman E. D., Battelli L., & Pascual-Leone A. (2005). Repetitive TMS over posterior STS disrupts perception of biological motion. *Vision Research,* 45, 2847–2853. 16039692 10.1016/j.visres.2005.05.027

[bib13] Grossman E. D., & Blake R. (1999). Perception of coherent motion, biological motion and form-from motion under dim-light conditions. *Vision Research,* 39, 3721–3727. 10746142 10.1016/s0042-6989(99)00084-x

[bib14] Grossman E. D., Donnelly M., Price R., Pickens D., Morgan V., Neighbor G., & Blake R. (2000). Brain areas involved in perception of biological motion. *Journal of Cognitive Neuroscience,* 12, 711–720. 11054914 10.1162/089892900562417

[bib15] Heeger D. J., & Jepson A. (1992). Subspace methods for recovering rigid motion I: Algorithm and implementation. *International Journal of Computer Vision,* 7, 95–117.

[bib16] Hirai M., Fukushima H., & Hiraki K. (2003). An event-related potentials study of biological motion perception in humans. *Neuroscience Letters,* 344, 41–44. 12781917 10.1016/s0304-3940(03)00413-0

[bib17] Johansson G. (1973). Visual perception of biological motion and a model for its analysis. *Perception & Psychophysics,* 14, 201–211.

[bib18] Krakowski A. I., Ross L. A., Snyder A. C., Sehatpour P., Kelly S. P., & Foxe J. J. (2011). The neurophysiology of human biological motion processing: A high-density electrical mapping study. *Neuroimage,* 56, 373–383. 21276862 10.1016/j.neuroimage.2011.01.058 PMC6589837

[bib19] Lange J., & Lappe M. (2006). A model of biological motion perception from configural form cues. *Journal of Neuroscience,* 26, 2894–2906. 16540566 10.1523/JNEUROSCI.4915-05.2006 PMC6673973

[bib20] Lappe M., Bremmer F., & van den Berg A. V. (1999). Perception of self-motion from visual flow. *Trends in Cognitive Sciences,* 3, 329–336. 10461195 10.1016/s1364-6613(99)01364-9

[bib21] Lappe M., & Rauschecker J. P. (1993). A neural network for the processing of optic flow from ego-motion in man and higher mammals. *Neural Computation,* 5, 374–391.

[bib22] Layton O. W., & Fajen B. R. (2016a). A neural model of MST and MT explains perceived object motion during self-motion. *Journal of Neuroscience,* 36, 8093–8102. 27488630 10.1523/JNEUROSCI.4593-15.2016 PMC6601960

[bib23] Layton O. W., & Fajen B. R. (2016b). Sources of bias in the perception of heading in the presence of moving objects: Object-based and border-based discrepancies. *Journal of Vision,* 16, 9, doi:10.1167/16.1.9. 26762278

[bib24] Layton O. W., Mingolla E., & Browning N. A. (2012). A motion pooling model of visually guided navigation explains human behavior in the presence of independently moving objects. *Journal of Vision,* 12(1):20, 1–19, doi:10.1167/12.1.20. 22275469

[bib25] Li L., Ni L., Lappe M., Niehorster D. C., & Sun Q. (2018). No special treatment of independent object motion for heading perception. *Journal of Vision,* 18(4)(19):19, 1–16, doi:10.1167/18.4.19. 29710309

[bib26] Li L., & Warren W. H. J. (2000). Perception of heading during rotation: Sufficiency of dense motion parallax and reference objects. *Vision Research,* 40, 3873–3894. 11090678 10.1016/s0042-6989(00)00196-6

[bib27] Longuet-Higgins H. C., & Prazdny K. (1980). The interpretation of a moving retinal image. *Proceedings of the Royal Society of London. Series B,* 208, 385–397. 10.1098/rspb.1980.0057 6106198

[bib28] Lu H. (2010). Structural processing in biological motion perception. *Journal of Vision,* 10:13, 1–13, doi:10.1167/10.12.13. 21047745

[bib29] Masselink J., & Lappe M. (2015). Translation and articulation in biological motion perception. *Journal of Vision,* 15, 10, doi:10.1167/15.11.10. 26270192

[bib30] Mayer K. M., Riddell H., & Lappe M. (2019). Concurrent processing of optic flow and biological motion. *Journal of Experimental Psychology,* 148, 1938–1952. 30829520 10.1037/xge0000568

[bib31] McKay L. S., Simmons D. R., McAleer P., & Pollick F. E. (2009). Contribution of configural information in a direction discrimination task: Evidence using a novel masking paradigm. *Vision Research,* 49, 2503–2508. 19682487 10.1016/j.visres.2009.08.008

[bib32] Michels L., Kleiser R., de Lussanet M. H. E., Seitz R. J., & Lappe M. (2009). Brain activity for peripheral biological motion in the posterior superior temporal gyrus and the fusiform gyrus: Dependence on visual hemifield and view orientation. *Neuroimage,* 45, 151–159. 19063979 10.1016/j.neuroimage.2008.10.063

[bib33] Morrone M. C., Tosetti M., Montanaro D., Fiorentini A., Cioni G., & Burr D. C. (2000). A cortical area that responds specifically to optic flow, revealed by fMRI. *Nature Neuroscience,* 3, 1322–1328. 11100154 10.1038/81860

[bib34] Perrone J. A. (1992). Model for the computation of self-motion in biological systems. *Journal of the Optical Society of America,* 9, 177–194. 1542059 10.1364/josaa.9.000177

[bib35] Pitzalis S., Sereno M. I., Committeri G., Fattori P., Galati G., Patria F., & Galletti C. (2010). Human v6: The medial motion area. *Cerebral Cortex,* 20, 411–424. 19502476 10.1093/cercor/bhp112 PMC2803738

[bib36] Raudies F., & Neumann H. (2012). A review and evaluation of methods estimating ego-motion. *Computer Vision and Image Understanding,* 116, 606–633.

[bib37] Reid R., Brooks A., Blair D., & van der Zwan R. (2009). Snap! recognising implicit actions in static point-light displays. *Perception,* 38, 613–616. 19522328 10.1068/p6320

[bib38] Riddell H., & Lappe M. (2017). Biological motion cues aid identification of self-motion from optic flow but not heading detection. *Journal of Vision,* 17, 19, doi:10.1167/17.12.19. 29090314

[bib39] Riddell H., & Lappe M. (2018). Heading through a crowd. *Psychological Science,* 29, 1504–1514. 30004826 10.1177/0956797618778498

[bib40] Riddell H., Li L., & Lappe M. (2019). Heading perception from optic flow in the presence of biological motion. *Journal of Vision,* 19(14):25, doi:10.1167/19.14.25. 31868898

[bib41] Royden C. S. (1996). A model using motion-oponent operators to compute translational and rotational heading parameters for a moving observer. *Abstracts - Society for Neuroscience,* 22, 718.

[bib42] Royden C. S., Banks M. S., & Crowell J. A. (1992). The perception of heading during eye movements. *Nature,* 360, 583–585. 1461280 10.1038/360583a0

[bib43] Royden C. S., Crowell J. A., & Banks M. S. (1994). Estimating heading during eye movements. *Vision Research,* 34, 3197–3214. 7975351 10.1016/0042-6989(94)90084-1

[bib44] Royden C. S., & Hildreth E. C. (1996). Human heading judgments in the presence of moving objects. *Perception & Psychophysics,* 58, 836–865. 8768180 10.3758/bf03205487

[bib45] Rushton S. K., Niehorster D. C., Warren P. A., & Li L. (2018). The primary role of flow processing in the identification of Scene-Relative object movement. *Journal of Neuroscience,* 38, 1737–1743. 29229707 10.1523/JNEUROSCI.3530-16.2017 PMC5815455

[bib46] Smith A. T., Wall M. B., Williams A. L., & Singh K. D. (2006). Sensitivity to optic flow in human cortical areas MT and MST. *European Journal of Neuroscience,* 23, 561–569. 16420463 10.1111/j.1460-9568.2005.04526.x

[bib47] Theusner S., de Lussanet M., & Lappe M. (2014). Action recognition by motion detection in posture space. *Journal of Neuroscience,* 34, 909–921. 24431449 10.1523/JNEUROSCI.2900-13.2014 PMC6608351

[bib48] Theusner S., de Lussanet M. H. E., & Lappe M. (2011). Adaptation to biological motion leads to a motion and a form aftereffect. *Perception & Psychophysics,* 73, 1843–1855. 10.3758/s13414-011-0133-7 21598067

[bib49] Thirkettle M., Scott-Samuel N. E., & Benton C. P. (2010). Form overshadows ‘opponent motion’ information in processing of biological motion from point light walker stimuli. *Vision Research,* 50, 118–126. 19909769 10.1016/j.visres.2009.10.021

[bib50] Thurman S. M., & Lu H. (2016). Revisiting the importance of common body motion in human action perception. *Perception & Psychophysics,* 78, 30–36. 10.3758/s13414-015-1031-1 26603043

[bib51] van den Berg A. V. (1992). Robustness of perception of heading from optic flow. *Vision Research,* 32, 1285–1296. 1455703 10.1016/0042-6989(92)90223-6

[bib52] Warren P. A., Rushton S. K., & Foulkes A. J. (2012). Does optic flow parsing depend on prior estimation of heading? *Journal of Vision,* 12, 7, doi:10.1167/12.11.8. PMC3826327 23064244

[bib53] Warren W. H., Blackwell A. W., Kurtz K. J., Hatsopoulos N. G., & Kalish M. L. (1991). On the sufficiency of the velocity field for perception of heading. *Biological Cybernetics,* 65, 311–320. 1742369 10.1007/BF00216964

[bib54] Warren W. H., & Hannon D. J. (1988). Direction of self-motion is perceived from optical flow. *Nature,* 336, 162–163.

[bib55] Warren W. H., & Hannon D. J. (1990). Eye movements and optical flow. *Journal of the Optical Society of America,* 7, 160–169. 2299447 10.1364/josaa.7.000160

[bib56] Warren W. H., Morris M. W., & Kalish M. (1988). Perception of translational heading from optical flow. *Journal of Experimental Psychology: Human Perception and Performance,* 14, 646–660. 2974874 10.1037//0096-1523.14.4.646

[bib57] Warren W. H., & Saunders J. A. (1995). Perceiving heading in the presence of moving objects. *Perception,* 24, 315–331. 7617432 10.1068/p240315

[bib58] Warren W. H. J. (1998). Visually controlled locomotion: 40 years later. *Ecological Psychology,* 10, 177–219.

[bib59] Yu X., Hou H., Spillmann L., & Gu Y. (2018). Causal evidence of motion signals in macaque middle temporal area Weighted-Pooled for global heading perception. *Cerebral Cortex,* 28(2), 612–624, 10.1093/cercor/bhw402. 28057722

